# Amplified effect of social vulnerability on health inequality regarding COVID-19 mortality in the USA: the mediating role of vaccination allocation

**DOI:** 10.1186/s12889-022-14592-w

**Published:** 2022-11-19

**Authors:** Ying Chen, Lanwei Zhang, Tenglong Li, Li Li

**Affiliations:** 1 Wisdom Lake Academy of Pharmacy, Xi’an Jiaotong-Liverpool University, Suzhou, 215123 China; 2 Department of Biology, School of Science, Xi’an Jiaotong-Liverpool University, Suzhou, 215123 China; 3 Department of Health and Environmental Sciences, School of Science, Xi’an Jiaotong-Liverpool University, Suzhou, 215123 China

**Keywords:** Social vulnerability, Health inequality, Mediation effect, COVID-19, Vaccination, Case fatality rate, Epidemiology, Ecological study, USA

## Abstract

**Background:**

Vaccination reduces the overall burden of COVID-19, while its allocation procedure may introduce additional health inequality, since populations characterized with certain social vulnerabilities have received less vaccination and been affected more by COVID-19. We used structural equation modeling to quantitatively evaluate the extent to which vaccination disparity would amplify health inequality, where it functioned as a mediator in the effect pathways from social vulnerabilities to COVID-19 mortality.

**Methods:**

We used USA nationwide county (*n* = 3112, 99% of the total) level data during 2021 in an ecological study design. Theme-specific rankings of social vulnerability index published by CDC (latest data of 2018, including socioeconomic status, household composition & disability, minority status & language, and housing type & transportation) were the exposure variables. Vaccination coverage rate (VCR) during 2021 published by CDC was the mediator variable, while COVID-19 case fatality rate (CFR) during 2021 published by John Hopkinson University, the outcome variable.

**Results:**

Greater vulnerabilities in socioeconomic status, household composition & disability, and minority status & language were inversely associated with VCR, together explaining 11.3% of the variance of VCR. Greater vulnerabilities in socioeconomic status and household composition & disability were positively associated with CFR, while VCR was inversely associated with CFR, together explaining 10.4% of the variance of CFR. Our mediation analysis, based on the mid-year data (30^th^ June 2021), found that 37.6% (mediation/total effect, 0.0014/0.0037), 10% (0.0003/0.0030) and 100% (0.0005/0.0005) of the effects in the pathways involving socioeconomic status, household composition & disability and minority status & language, respectively, were mediated by VCR. As a whole, the mediation effect significantly counted for 30.6% of COVID-19 CFR disparity. Such a mediation effect was seen throughout 2021, with proportions ranging from 12 to 32%.

**Conclusions:**

Allocation of COVID-19 vaccination in the USA during 2021 led to additional inequality with respect to COVID-19 mortality. Viable public health interventions should be taken to guarantee an equitable deployment of healthcare recourses across different population groups.

**Supplementary Information:**

The online version contains supplementary material available at 10.1186/s12889-022-14592-w.

## Background

Health disparities due to sociodemographic factors, under the shadow of COVID-19, reveal the inadequacy of current public health strategies in achieving ‘optimal health for all’ [1]. The problem becomes more pronounced in developed countries where healthcare resources are relatively abundant. Communities with lower socioeconomic status have higher COVID-19 morbidity and mortality, especially in people of minority, those of working class with lower education levels, poverty, poor housing, low household incomes, overcrowded living, food insecurity, lacking health insurance and speaking a language other than the national language [2–20]. These communities require necessary interventions for a better deployment of healthcare resources, ensuring equity in access to prevention and care services across different population groups during the pandemic [1, 21].

As an effective way to reduce new and more severe cases [22–25], vaccination provides hope that the pandemic may end and normalize life by reducing new infections and clinical severity. The WHO Europe Health 2020 policy framework prioritizes equitable access to vaccination [26]. The USA has a framework for equitable allocation of COVID-19 vaccine [27, 28]. However, a growing body of literature has shown that lower COVID-19 vaccination coverage rates (VCRs) are common in social vulnerable populations [28–38]. The reasons are multifold. Vaccine hesitancy may be one of the barriers [38–40]. Furthermore, structural barriers, such as language, transportation, computer/internet access, immigrant status, and long distances to local healthcare facilities, may also play important roles [35, 41]. Inequality in vaccination coverage is a particular concern, especially since socially vulnerable populations already have been disproportionately affected by COVID-19 [2–20].

COVID-19 vaccination is vital in the reduction of COVID-19 disease burden [42]. However, vaccination implementation may exacerbate health inequality since communities with certain social vulnerabilities also have lower VCRs. We, therefore hypothesized, that the association of social vulnerability status with COVID-19 outcomes would be amplified by vaccination implementation, where vaccination inequality plays a significant mediator role. Identifying this issue would promote health equality during the current pandemic.

## Methods

Our study uses structural equation modeling to evaluate the mediation effect potentially existing in the pathway between social vulnerability and COVID-19 mortality, based on the county-level USA data during 2021.

### Data and study design

We used data from USA counties (or county equivalent) as sample units, with information in three areas: 1) the social vulnerability index (year 2018), 2) COVID-19 vaccination coverage (year 2021), and 3) COVID-19 mortality (year 2021).

The social vulnerability index (SVI, from the USA Centers for Disease Control and Prevention (CDC), 2018), which was created by the Geospatial research, Analysis & Services Program under the Agency for Toxic Substances and Disease Registry [43]. The SVI is used by health authorities and emergency response planners identify and map the communities that need support before, during, and after a hazardous event. The SVI serves as an indicator of the relative vulnerability of every USA census tract, and it ranks the tracts in terms of 15 social factors and further groups them into four themes [44]. Percentile rankings are available for 15 individual factors. Theme-specific ranking is generated by summing the percentiles of the factors in each theme and ordering the summed percentiles. Percentile ranking is set in the range from 0 to 1, with larger values demonstrating a greater vulnerability [44]. County-level characteristics on the four SVI themes (i.e. socioeconomic status, household composition & disability, minority status & language, and housing type & transportation) are shown in Supplementary Fig. 1.

**Fig. 1:**
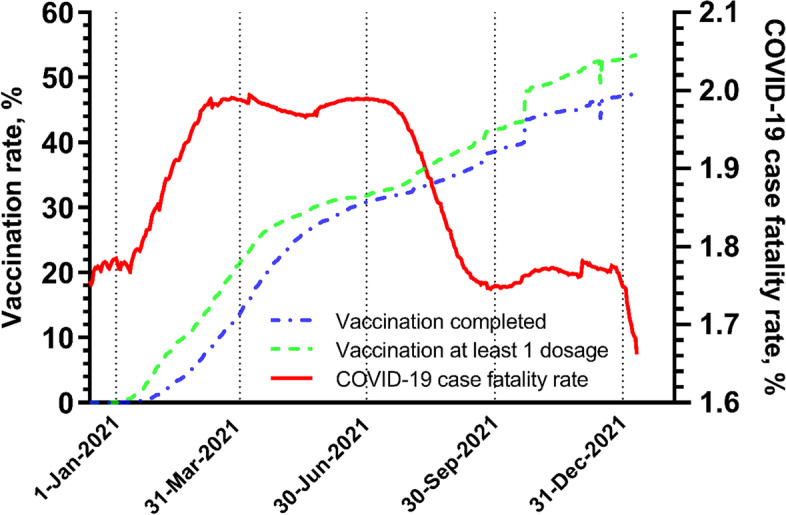
The vaccination coverage rate and case fatality rate of COVID-19 in USA during 2021

Data on the VCR of COVID-19 was extracted from the database provided by the CDC [45]. It collects reliable data (e.g. proportion who are fully vaccinated, and proportion with at least one dose) at county level for monitoring daily progress in COVID-19 vaccination (Fig. 1).

The case fatality rate (CFR) of COVID-19 (i.e. mortality rate per number of cases) in each USA county was accessed from the John Hopkins COVID-19 interactive map, a web-based dashboard to track COVID-19 in real-time on a daily basis [46, 47]. The CFR was computed based on the numbers of reported cases and deaths extracted from the interactive map [46]. County-level CFR during 2021 is shown in Fig. 1.

Our study sample consisted of 3112 counties (out of the total 3142, 99.0%), for which all study variables were complete. The 30 counties with missing data which were excluded from our analysis were: Beaver, Box Elder, Cache, Carbon, Daggett, Duchesne, Emery, Garfield, Grand, Iron, Juab, Kane, Millard, Morgan, Piute, Rich, Sanpete, Sevier, Uintah, Washington, Wayne and Weber (in Utah), Bristol Bay, Hoonah-Angoon, Kusilvak, and Valdez-Cordova (in Alaska), Kalawao (in Hawaii), Dukes and Nantucket (in Massachusetts), and Rio Arriba (in New Mexico).

### Statistical analysis

We used descriptive statistics of the studied variables (including SVI, VCR and CFR) at county level to analyse our data. For each SVI theme, we compared counties at the top half (more vulnerable, *n* = 1556) with those at the bottom half (less vulnerable, *n* = 1556). VCF and CFR were continuous variables within a proportional range. A simple linear regression model was first used to infer the initial association between each SVI theme and VCR. A multivariable linear regression model with backward selection, was then used to identify the final list of SVI themes associated with VCR. Similar process was done for the relationship between SVI themes and CFR. The association between VCR and CFR was also analyzed.

We hypothesized that the associations of SVI themes with CFR were mediated, at least partially, by VCR. A single-mediator model was then developed to determine the effect of SVI (‘X’, as exposure) on CFR (‘Y’, as outcome), as well as the mediating role of VCR (‘M’, as mediator) in the effect pathways. To estimate the effects, a series of liner regression models for constructing the structural equation modeling was carried out. Effects from ‘X’ to ‘M’ and from ‘M’ to ‘Y’ were marked as *a* and *b,* respectively. Direct effect from ‘X’ to ‘Y’ was marked as *c’*. The total effect *c* = *c’* + *a* × *b.*

Associations based on the point of mid-year (30^th^ June 2021) were reported as the main results. Validation analyses in order to question whether the findings were robust over time were conducted at the other time points on a monthly basis. Results for 31^st^ March, 30^th^ September and 31^st^ December were also reported in a detailed way. The trends of effects during 2021 were summarized based on mediation analysis.

We set the level of statistical significance as 0.001 in all analyses, in order to report conservatively significant estimates of associations discovered over time. The statistical analyses were carried out in STATA 15.

## Results

On 30^th^ June 2021, based on the county-level data, on average 30.8% of the USA population was fully vaccinated, while the case fatality rate (CFR) was 2.0% (Fig. 1, and Supplementary Table 1).

Greater vulnerabilities in socioeconomic status, household composition & disability, and minority status & language were found to be inversely associated with vaccination coverage rate (VCR) in simple regression models (left, Table 1), and these associations remained significant after adjusting for each other in the final multivariable regression analysis. Together, the four SVI themes explained 11.3% of the variance of VCR (right, Table 1).

**Table 1:** Association of social vulnerability index (SVI) with vaccination coverage rate (VCR) of COVID-19 (fully vaccinated, based on the data of 30^th^ June 2021)

	Simple regression analyses		Multiple regression analysis	
	Regression coefficient (99.9% confidence intervals)	R^2^ (for individual variables)	Regression coefficient (99.9% confidence intervals)	R^2^ (for the whole model)
Individual variable				
Socioeconomic status (greater vulnerability)	-0.0995 (-0.1167, -0.0822)	0.1035	-0.0851 (-0.1051, -0.0652)	-
Household composition & disability (greater vulnerability)	-0.0621 (-0.0799, -0.0442)	0.0401	-0.0199 (-0.0395, -0.0002)	-
Minority status & language (greater vulnerability)	-0.0429 (-0.0610, -0.0248)	0.0190	-0.0278 (-0.0452, -0.0103)	-
Housing type & transportation (greater vulnerability)	-0.0083 (-0.0265, 0.0099)	-	-	-
Model	-	-	-	0.1134

Greater vulnerabilities in socioeconomic status and household composition & disability were positively associated with CFR in simple regression models (Table 2, left). VCR was also associated in an inverse way (Table 2, left). In the multivariable regression model containing these three factors, each of those associations remained significant (Table 2, right). Together, these three factors explained 10.4% of the variance of CFR (Table 2, right).

**Table 2:** Association of social vulnerability index (SVI) and vaccination coverage rate (VCR) of COVID-19 with case fatality rate (CFR) of COVID-19 (fully vaccinated, based on the data of 30^th^ June 2021)

	Simple regression analyses		Multiple regression analysis	
	Regression coefficient (99.9% confidence intervals)	R^2^ (for individual variables)	Regression coefficient (99.9% confidence intervals)	R^2^ (for the whole model)
Individual variable				
Social vulnerability index				
Socioeconomic status (greater vulnerability)	0.0048 (0.0037, 0.0060)	0.0575	0.0023 (0.0010, 0.0036)	-
Household composition & disability (greater vulnerability)	0.0045 (0.0034, 0.0057)	0.0512	0.0027 (0.0014, 0.0039)	-
Minority status & language (greater vulnerability)	0.0003 (-0.0009, 0.0014)	-	-	-
Housing type & transportation (greater vulnerability)	0.0005 (-0.0007, 0.0017)	-	-	-
Vaccination coverage rate of COVID-19	-0.0163 (-0.0200, -0.0126)	0.0631	-0.0122 (-0.0160, -0.0084)	-
Model	-	-	-	0.1044

**Fig. 2:**
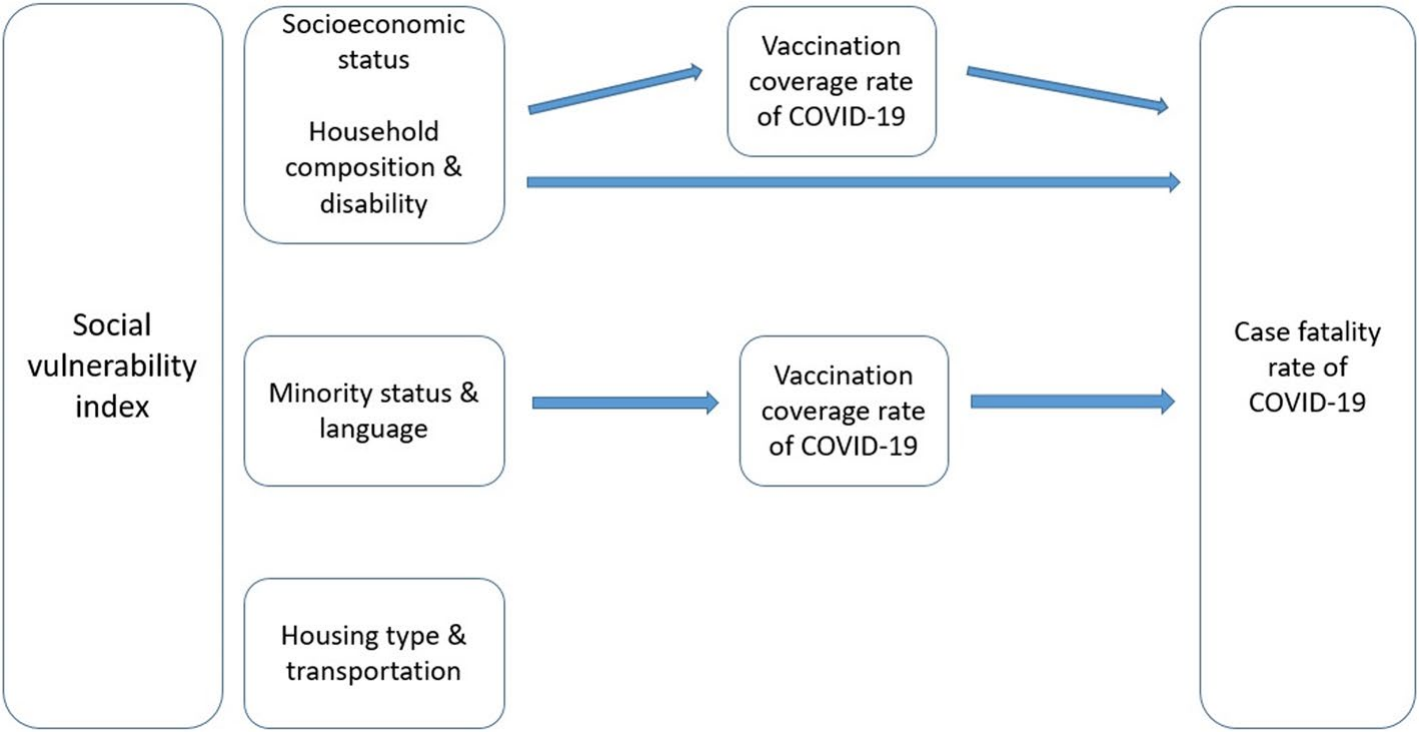
Vaccination coverage rate as a mediator in the impact pathway between certain themes of social vulnerability index and case fatality rate of COVID-19

These results require further investigation of a possible mediation effect using the structural equation modeling, and a proposed diagram of impact pathways shown in Fig. 2.

To evaluate the mediation effect of VCR, we defined the effects of all pathways (including path *a*, path *b*, path *c’*, and path *c*) based on the data of 30^th^ June 2021 (Table 3). Both socioeconomic status and household composition & disability had direct effects (path *c’*) on CFR of COVID-19, after adjusting for VCR. Compared with the counties ranked in the less vulnerable half those in the more vulnerable half in the themes of socioeconomic status and household composition & disability had 0.23% and 0.27% of increases in CFR, respectively (Table 3). Furthermore, via the mediation pathway (path *a* × *b*) by VCR, these two social vulnerability factors had indirect effects on CFR (0.14% and 0.03%, respectively, Table 3). Thus, the total effects (path *c*) of socioeconomic status and household composition & disability on CFR were 0.37% and 0.30%, respectively (Table 3). For minority status & language, there was no significant direct effect (path *c’*), but a small effect (0.05%) was observed in the mediation pathway (path *a* × *b*) (Table 3).

**Table 3:** Quantification of mediation effects (based on the data of 30^th^ June 2021)

	By social vulnerability index domain			Overall
	Socioeconomic status	Household composition & disability	Minority status & language	
Path *a*: (X > M)	-0.0851	-0.0199	-0.0278	-
Path *b*: (M > Y)	-0.0163	-0.0163	-0.0163	-
Indirect effect (*a* × *b*: X > M > Y)	0.0014	0.0003	0.0005	0.0022
Direct effect (Path *c’*: X > Y adjusted for M)	0.0023	0.0027	-	0.0050
Total effect (Path *c*: X > Y)	0.0037	0.0030	0.0005	0.0072
Mediated (*a* × *b/c*), %	37.8	10.0	100.0	30.6

Overall, based on the data of 30^th^ June 2021, three SVI themes (i.e. socioeconomic status, household composition & disability, and minority status & language) were found to be associated with CFR, where VCR played a mediator role. Approximately 30.6% of the total effect can be attributed to the mediation effect of VCR (Table 3).

**Fig. 3:**
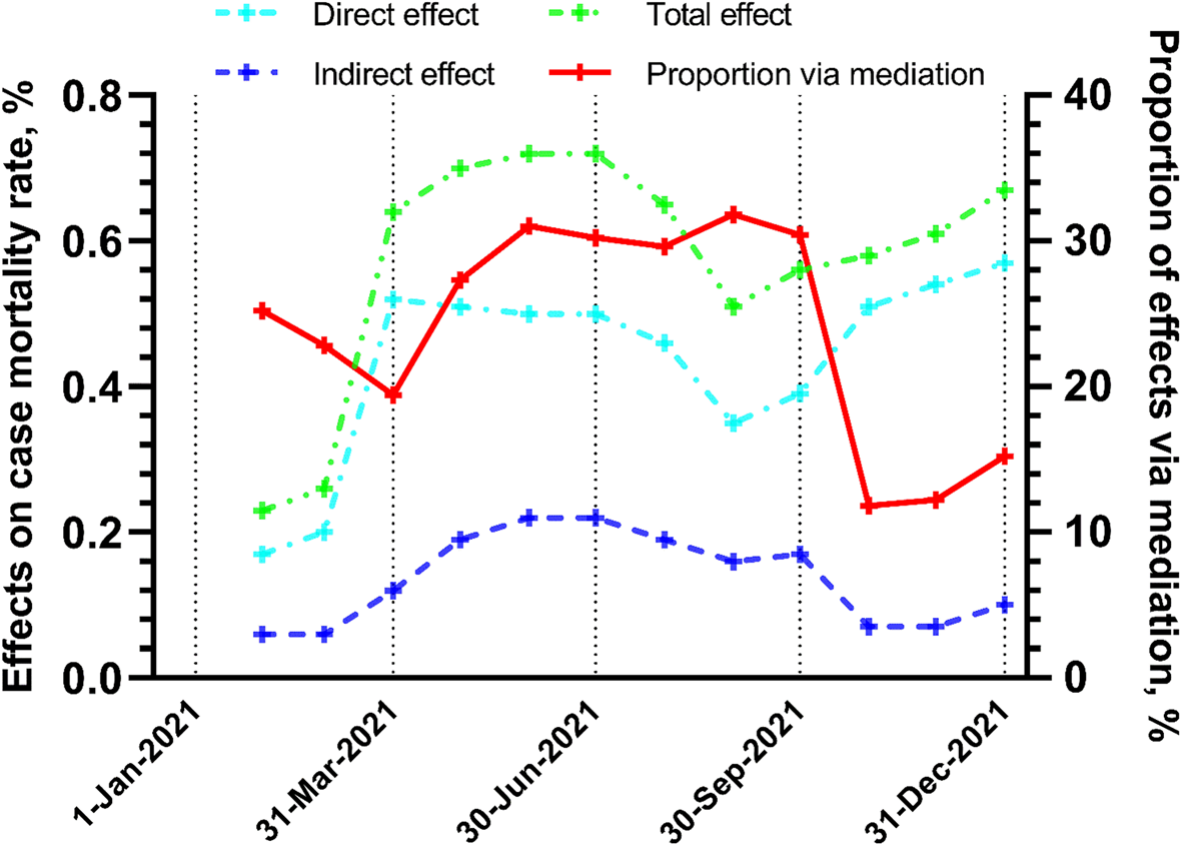
The direct, mediation (indirect) and total effects calculated monthly over the year of 2021

The mediation effect of VCR could be confirmed throughout 2021, although the estimated effect sizes varied from time to time and they tended to be smaller in the latter part of 2021 (Fig. 3, and Supplementary Tables 2, 3 and 4).

## Discussion

The COVID-19 pandemic continues to be a global crisis in public health since the initial outbreak in 2019. Previous research has shown that populations with social vulnerability are associated with severer disease outcomes [2–19]. We hypothesized that, after the introduction of COVID-19 vaccination, such an association would be amplified since socially vulnerable people are less likely to be vaccinated [28–38]. By using mediation analysis, our results confirmed our hypothesis showing that inequality of vaccination allocation was the mediator of the social vulnerability-health inequality association.

We used the theme-specific social vulnerability index (SVI) which included four themes: socioeconomic status, household composition & disability, minority status & language, and housing type & transportation to investigate their relationships with mortality rate per number of cases of COVID-19. Our results were consistent with previous research which reported that incidence and mortality rates of COVID-19 were disproportionately higher in the USA counties with greater social vulnerability [18, 48]. The magnitudes of impacts varied significantly across different themes of social vulnerability. Using county level data for 2020, Karaye and Horney showed that COVID-19 incidence was associated with household composition & disability, minority status & language and housing type & transportation, and the theme of minority status & language had the strongest impact on COVID-19 incidence [48]. Our results identified socioeconomic status and household composition & disability as the two most influential themes for COVID-19 mortality.

Vaccination is helpful for reducing the overall burden of COVID-19 [42]. However, disparities in vaccination by social vulnerability have been recorded since the beginning of the USA’s COVID-19 vaccination campaign in December 2020 [28–37, 49]. We analysed and reported the data from the beginning of vaccination campaign, to depict the timely trend in vaccination disparities. Our mediation analysis showed that additional effects of social vulnerabilities on case fatality rate (CFR) was mediated by vaccination coverage rate (VCR). Across the four social vulnerability themes, the largest mediation effect was found in the ‘socioeconomic status (lower socioeconomic status – lower vaccination coverage – higher case fatality rate)’ pathway, followed by the pathways of ‘household composition & disability’ and ‘minority status & language’. Interestingly, the effect of minority status & language on COVID-19 CFR was mainly attributed to mediation as no direct effect was detected, revealing the essential role of VCR in this pathway.

Health inequalities often surface when the structure of a society is affected by a new disease or disease prevention and control measures which benefit only certain communities [4]. People with lower socioeconomic status are less likely to undertake social distancing since they are typically identified as having jobs as essential workers or labourers [50]. They tend to live and work in crowded places, having less protection against COVID-19 [50]. These people may take more time to understand the disease and ways to protect themselves, especial due to lower educational levels [51, 52]. Communities with more econmonic resource and better infrastrucutre are likely better prepared for COVID-19 prevention and care [15, 16, 20, 28, 37]. Communities with social disadvantage were assoicated with fewer beds per number of residents in New York City [53]. A Brazilan study showed that the level of health-system readiness and response to COVID-19 was largely dependent on the socieconomic status of individual communities across the country [54]. Especially, when novel health interventions such as vaccines are implemented in limited supply, it is resulted in competition among individuals or communities, favoring those of higher socioeconomic status [28, 37].

The UK Scientific Advisory Group for Emergencies identified four barriers to COVID-19 vaccine uptake among ethnic minority groups: inconvenience and access barriers (e.g. cost, time and distance to access vaccine), context and socio-demographic variation (e.g. lower uptake among people with low levels of education), low trust and confidence (in vaccine efficacy and safety), and lower perception of disease risk [55]. Similarly, those aforementioned structural barriers (or similar barriers), together with vaccine hesitancy, led to the low vaccine uptake among ethnic minority groups in the USA [41, 56]. By September 2021, over 75% of adults in the USA had received at least one dose of COVID-19 vaccine, however, the proportions of vaccinated adults were less than 20% in Hispanic/Latino and black populations [41]. We have shown that insufficient English language skills and mobility restriction (e.g. disability) are also important barriers. In order to promote the vaccination equity in society, the public should acknowledge that sociocultural tailored approaches are needed to engage particular groups and build trust [40]. These path-dependency-breaking measures can be coupled with optimized vaccine accesses, such as optimized spatial arrangement of vaccination venues in socially disadvantaged neighborhoods, to promote vaccine uptake among social vulnerable groups [40].

Link and Phelan developed the theory of ‘fundamental causes’ to explain the relationship between social conditions and health inequalities [57, 58]. In this theory, diseases transition through four phases over a period: 1) natural mortality, characterized by no knowledge about risk factors, preventions, or treatments for a disease in a population; 2) producing inequalities, characterized by unequal diffusion of innovations; 3) reducing inequalities, characterized by increased access to health knowledge; and 4) reduced mortality/disease elimination, characterized by widely available prevention and effective treatment [59]. We discovered a significant mediator role of vaccination coverage rate **(**VCR) throughout 2021, however, the mediation effect tended to be smaller after September. Our observation to some extent supports this theory. We suggest that the initiation of vaccination, as a new protective method from COVID-19 death, produces additional inequality on top of the existing difference in social vulnerability. After a period of time, due to increased access to vaccine (e.g. increased vaccine supply), the additional inequality starts to diminish. However, this evolving trend of mediation effect during 2021 may also be influenced by the health determinants of COVID-19 CFR, as these factors (including virus variants) were dynamic over time [3]. We suggest that longitudinal monitoring data should be used to record and interpret these temporal trends.

To develop adaptive public health countermeasures, it is helpful to identify any tipping point or time lag between the intervention measures e.g. VCR and the health responses e.g. COVID-19 CFR. For instance, when the full vaccination rate reached 30% in July, the CFR started to drop from 2.0%, and leveled off at 1.8% from September until December (Fig. 1). Subsequently, a turning point of vaccination’s mediation effect occurred around September, followed by a decreasing mediation effect (measured by the proportion of mediation effect out of total effect) from 35 to 12% in two months (Fig. 3). The rapid shift in public health responses requires speedy public health workforce action to take place in narrow time window [60]. Sentinel studies may be useful to interpret system change signals and identify priorities for actions to ensure social equity of healthcare resource [49].

The COVID-19 pandemic may further exert impact on social and health inequalities in many aspects and in the long-term [61]. COVID-19 is known to occur at increased risk and result in more severe outcomes for individuals who have multiple comorbidities [61]. Furthermore, there is growing concern about the post-COVID-19 syndrome which is more likely to affect those who were already disadvantaged [62].

Our study does have a few limitations. Firstly, we focused only on social vulnerability, vaccination allocation and COVID-19 mortality, but additional information that may have influenced the studied relationships, such as other regional specific county factors was not included. Secondly, we used an ecological study design, so possible bias due to factors such as migration may have an impact. However, since the COVID-19 pandemic is new, such bias should be minor within a relative short duration. Thirdly, we were unable to rule out potential reporting bias that may exist across different counties, although the data we used may be considered to be of a quality [43–46].

We have demonstrated that by using mediation analysis based on USA county level data during 2021, we discovered an amplified effect of social vulnerability in health inequality on COVID-19 mortality, which may be attributed to the mediation effect of vaccination allocation across different counties.

## Conclusions

We have demonstrated that health inequality and its driving factors should be explored by public health policymakers, practitioners, and clinicians. Efforts should be made to narrow the health disparities by identifying and supporting socially vulnerable populations. Furthermore, when implementing a novel preventive measure such as vaccines, it is important to ensure equitable access to different social groups, thus preventing further amplification in health inequality. We suggest that future work should be done at the community level, possibly with targeted interventions, to develop viable countermeasures to any amplified health system inequality which may arise.

## Supplementary Information

**Additional file 1:** **Supplementary Figure 1.** Graphical maps of the Social Vulnerability Index 2018 by themes at the county level in USA. **Supplementary Table 1.** Vaccination coverage rate of COVID-19, and case fatality rate of COVID-19 at the county level of USA on 31^st^March, 30^th^ June, 30^th^September, and 31^st^ December 2021. **Supplementary Table 2.** Association of social vulnerability index with vaccination coverage rate of COVID-19 (fully vaccinated, based on the data of 31^st^ March 2021, 30^th^ September 2021, and 31^st^ December 2021). **Supplementary Table 3.** Association of social vulnerability index and vaccination coverage rate of COVID-19 with case fatality rate of COVID-19 (fully vaccinated, based on the data of 31^st^ March 2021, 30^th^ September 2021, and 31^st^ December 2021). **Supplementary Table 4.** Quantification of medication effects (based on the data of 30^th^ March 2021, 31^st^ September 2021, and 31^st^ December 2021).

## Data Availability

SVI (2018), created by the Geospatial research, Analysis & Services Program under the Agency for Toxic Substances and Disease Registry, was publicly accessible at https://www.atsdr.cdc.gov/placeandhealth/svi/documentation/SVI_documentation_2018.html. Data on the VCR of COVID-19 was extracted from the database provided by the USA CDC at https://data.cdc.gov/Vaccinations/COVID-19-Vaccinations-in-the-United-States-County/8xkx-amqh. CFR of COVID-19 in each USA county was obtained from the University of John Hopkinson, a web-based dashboard to track COVID-19 in real-time on a daily basis, at https://github.com/CSSEGISandData/COVID-19.

## Abbreviations

CDC: Centers for Disease Control and Prevention
CFR: Case Fatality Rate
COVID-19: Coronavirus Disease 2019
SVI: Social Vulnerability Index
USA: United States of America
VCR: Vaccination Coverage Rate
WHO: World Health Organization
