# Supplementary material for: Amplified effect of social vulnerability on health inequality regarding COVID-19 mortality in the USA: the mediating role of vaccination allocation
Source: BMC Public Health. 2022 Nov 19;22:2131. doi: 10.1186/s12889-022-14592-w (PMC9675971; doi:10.1186/s12889-022-14592-w)
Supplement: Supplementary file 1 — Additional file 1: Supplementary Figure 1. Graphical maps of the Social Vulnerability Index 2018 by themes at the county level in USA. Supplementary Table 1. Vaccination coverage rate of COVID-19, and case fatality rate of COVID-19 at the county level of USA on 31stMarch, 30th June, 30thSeptember, and 31st December 2021. Supplementary Table 2. Association of social vulnerability index with vaccination coverage rate of COVID-19 (fully vaccinated, based on the data of 31st March 2021, 30th September 2021, and 31st December 2021). Supplementary Table 3. Association of social vulnerability index and vaccination coverage rate of COVID-19 with case fatality rate of COVID-19 (fully vaccinated, based on the data of 31st March 2021, 30th September 2021, and 31st December 2021). Supplementary Table 4. Quantification of medication effects (based on the data of 30th March 2021, 31st September 2021, and 31st December 2021). [file 12889_2022_14592_MOESM1_ESM.docx]

**Amplified effect of social vulnerability on health inequality regarding COVID-19 mortality: The mediating role of vaccination allocation**

**Supplementary Figure 1. Graphical maps of the Social Vulnerability Index 2018 by themes at the county level in USA**


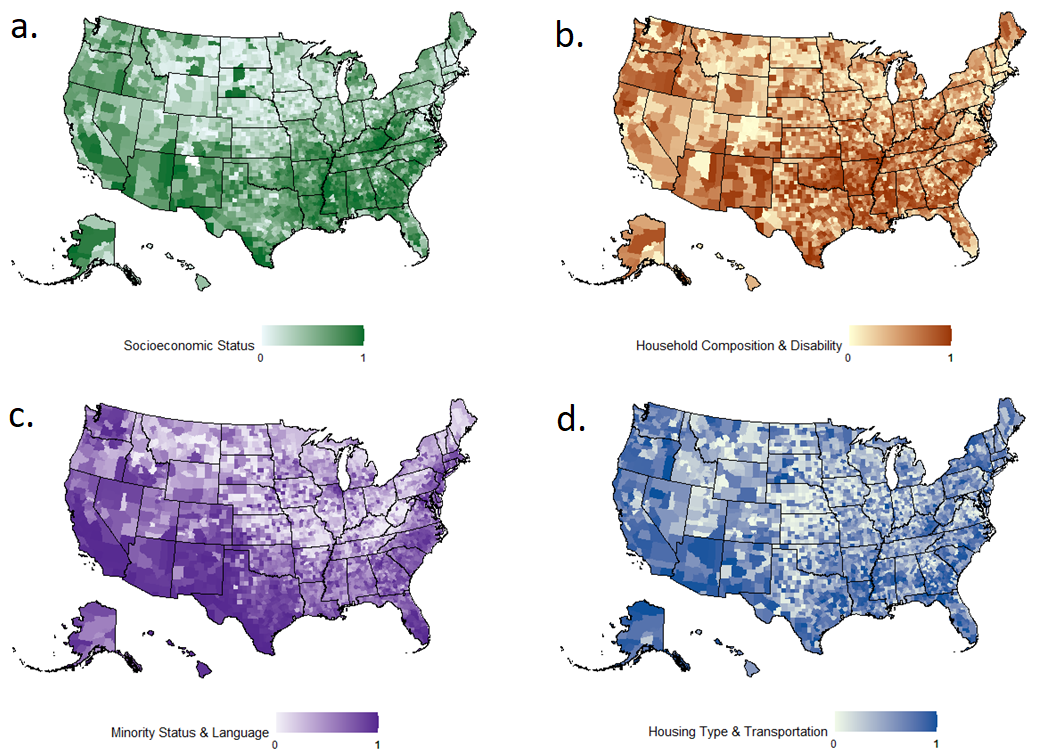


**Supplementary Table 1. Vaccination coverage rate of COVID-19, and case fatality rate of COVID-19 at the county level of USA on 31^st^ March, 30^th^ June, 30^th^ September, and 31^st^ December 2021**

|  | Mean  (Standard Deviation) |
| --- | --- |
| Vaccination coverage rate of COVID-19 |  |
| Fully vaccinated, on 31^st^ March 2021 | 0.137 (0.075) |
| Fully vaccinated, on 30^th^ June 2021 | 0.308 (0.154) |
| Fully vaccinated, on 30^th^ September 2021 | 0.386 (0.167) |
| Fully vaccinated, on 31^st^ December 2021 | 0.472 (0.131) |
| Case fatality rate of COVID-19 |  |
| Rate on 31^st^ March 2021 | 0.020 (0.010) |
| Rate on 30^th^ June 2021 | 0.020 (0.010) |
| Rate on 30^th^ September 2021 | 0.017 (0.009) |
| Rate on 31^st^ December 2021 | 0.017 (0.008) |

**Supplementary Table 2. Association of social vulnerability index with vaccination coverage rate of COVID-19 (fully vaccinated, based on the data of 31^st^ March 2021, 30^th^ September 2021, and 31^st^ December 2021)**

| 31^st^ March 2021 | Simple regression analyses | | Multiple regression analysis | |
| --- | --- | --- | --- | --- |
|  | Regression coefficient (99.9% confidence intervals) | R^2^  (for individual variables) | Regression coefficient  (99.9% confidence intervals) | R^2^  (for the whole model) |
| Individual variable |  |  |  |  |
| Socioeconomic status | -0.0318 (-0.0405, -0.0231) | 0.0441 | -0.0293 (-0.0392, -0.0193) | - |
| Household composition & disability | -0.0107 (-0.0196, -0.0018) | 0.0047 | 0.0045 (-0.0053, 0.0143) | - |
| Minority status & language | -0.0325 (-0.0412, -0.0238) | 0.0461 | -0.0277 (-0.0364, -0.0190) | - |
| Housing type & transportation | -0.0076 (-0.0165, 0.0013) | - | - | - |
| Model | - | - | - | 0.0774 |
| 30^th^ September 2021 | Simple regression analyses | | Multiple regression analysis | |
|  | Regression coefficient (99.9% confidence intervals) | R^2^  (for individual variables) | Regression coefficient  (99.9% confidence intervals) | R^2^  (for the whole model) |
| Individual variable |  |  |  |  |
| Socioeconomic status | -0.0992 (-0.1179, -0.0804) | 0.0883 | -0.0847 (-0.1065, -0.0630) | - |
| Household composition & disability | -0.0660 (-0.0853, -0.0467) | 0.0389 | -0.0244 (-0.0458, -0.0029) | - |
| Minority status & language | -0.0307 (-0.0503, -0.0111) | 0.0082 | -0.0155 (-0.0345, 0.0036) | - |
| Housing type & transportation | -0.0050 (-0.0246, 0.0147) | - |  | - |
| Model | - | - | - | 0.0936 |
| 31^st^ December 2021 | Simple regression analyses | | Multiple regression analysis | |
|  | Regression coefficient (99.9% confidence intervals) | R^2^  (for individual variables) | Regression coefficient  (99.9% confidence intervals) | R^2^  (for the whole model) |
| Individual variable |  |  |  |  |
| Socioeconomic status | -0.0729 (-0.0878, -0.0580) | 0.0767 | -0.0676 (-0.0847, -0.0506) | - |
| Household composition & disability | -0.0551 (-0.0703, -0.0399) | 0.0437 | -0.0236 (-0.0404, -0.0068) | - |
| Minority status & language | 0.0242 (0.0087, 0.0396) | 0.0081 | 0.0364 (0.0215, 0.0514) | - |
| Housing type & transportation | 0.0152 (-0.0003, 0.0307) | - | - | - |
| Model | - | - | - | 0.1022 |

**Supplementary Table 3. Association of social vulnerability index and vaccination coverage rate of COVID-19 with case fatality rate of COVID-19 (fully vaccinated, based on the data of 31^st^ March 2021, 30^th^ September 2021, and 31^st^ December 2021)**

| 31^st^ March 2021 | Simple regression analyses | | Multiple regression analysis | |
| --- | --- | --- | --- | --- |
|  | Regression coefficient (99.9% confidence intervals) | R^2^  (for individual variables) | Regression coefficient  (99.9% confidence intervals) | R^2^  (for the whole model) |
| Individual variable |  |  |  |  |
| Social vulnerability index |  |  |  |  |
| Socioeconomic status | 0.0043 (0.0031, 0.0055) | 0.0424 | 0.0023 (0.0009, 0.0037) | - |
| Household composition & disability | 0.0042 (0.0030, 0.0054) | 0.0406 | 0.0029 (0.0015, 0.0043) | - |
| Minority status & language | 0.0001 (-0.0012, 0.0013) | - | - | - |
| Housing type & transportation | 0.0004 (-0.0009, 0.0016) | - | - | - |
| Vaccination coverage rate of COVID-19 | -0.0238 (-0.0319, -0.0158) | 0.0294 | -0.0193 (-0.0273, -0.0112) | - |
| Model | - | - | - | 0.0739 |
| 30^th^ September 2021 | Simple regression analyses | | Multiple regression analysis | |
|  | Regression coefficient (99.9% confidence intervals) | R^2^  (for individual variables) | Regression coefficient  (99.9% confidence intervals) | R^2^  (for the whole model) |
| Individual variable |  |  |  |  |
| Social vulnerability index |  |  |  |  |
| Socioeconomic status | 0.0038 (0.0028, 0.0048) | 0.0490 | 0.0018 (0.0006, 0.0029) | - |
| Household composition & disability | 0.0036 (0.0026, 0.0046) | 0.0437 | 0.0021 (0.0009, 0.0032) | - |
| Minority status & language | 0.0003 (-0.0007, 0.0013) | - | - | - |
| Housing type & transportation | 0.0002 (-0.0008, 0.0012) | - | - | - |
| Vaccination coverage rate of COVID-19 | -0.0137 (-0.0167, -0.0107) | 0.0693 | -0.0109 (-0.0139, -0.0078) | - |
| Model | - | - | - | 0.1020 |
| 31^st^ December 2021 | Simple regression analyses | | Multiple regression analysis | |
|  | Regression coefficient (99.9% confidence intervals) | R^2^  (for individual variables) | Regression coefficient  (99.9% confidence intervals) | R^2^  (for the whole model) |
| Individual variable |  |  |  |  |
| Social vulnerability index |  |  |  |  |
| Socioeconomic status | 0.0054 (0.0045, 0.0064) | 0.1122 | 0.0033 (0.0023, 0.0044) | - |
| Household composition & disability | 0.0047 (0.0038, 0.0056) | 0.0839 | 0.0024 (0.0014, 0.0034) | - |
| Minority status & language | 0.0002 (-0.0007, 0.0012) | - | - | - |
| Housing type & transportation | 0.0007 (-0.0003, 0.0016) | - | - | - |
| Vaccination coverage rate of COVID-19 | -0.0186 (-0.0221, -0.0151) | 0.0904 | -0.0132 (-0.0167, -0.0097) | - |
| Model | - | - | - | 0.1747 |

**Supplementary Table 4. Quantification of medication effects (based on the data of 30^th^ March 2021, 31^st^ September 2021, and 31^st^ December 2021)**

| 31^st^ March 2021 | By social vulnerability index domain | | | Overall |
| --- | --- | --- | --- | --- |
|  | Socioeconomic status | Household composition & disability | Minority status & language |  |
| Path a: (X > M) | -0.0293 | 0.0045 | -0.0277 | - |
| Path b: (M > Y) | 0.0238 | -0.0238 | -0.0238 | - |
| Indirect effect (a×b: X > M > Y) | 0.0007 | -0.0001 | 0.0007 | 0.0012 |
| Direct effect (Path c’: X > Y adjusted for M) | 0.0023 | 0.0029 | - | 0.0052 |
| Total effect (Path c: X > Y) | 0.0030 | 0.0028 | 0.0007 | 0.0064 |
| Mediated (a×b/c), % | 23.3 | -3.8 | 100.0 | 19.4 |
|  |  |  |  |  |
| 30^th^ September 2021 | By social vulnerability index domain | | | Overall |
|  | Socioeconomic status | Household composition & disability | Minority status & language |  |
| Path a: (X > M) | -0.0847 | -0.0244 | -0.0155 | - |
| Path b: (M > Y) | -0.0137 | -0.0137 | -0.0137 | - |
| Indirect effect (a×b: X > M > Y) | 0.0012 | 0.0003 | 0.0002 | 0.0017 |
| Direct effect (Path c’: X > Y adjusted for M) | 0.0018 | 0.0021 | - | 0.0039 |
| Total effect (Path c: X > Y) | 0.0030 | 0.0024 | 0.0002 | 0.0056 |
| Mediated (a×b/c), % | 39.2 | 13.7 | 100.0 | 30.4 |
| 31^st^ December 2021 | By social vulnerability index domain | | | Overall |
|  | Socioeconomic status | Household composition & disability | Minority status & language |  |
| Path a: (X > M) | -0.0676 | -0.0236 | 0.0364 | - |
| Path b: (M > Y) | -0.0186 | -0.0186 | -0.0186 | - |
| Indirect effect (a×b: X > M > Y) | 0.0013 | 0.0004 | -0.0007 | 0.0010 |
| Direct effect (Path c’: X > Y adjusted for M) | 0.0033 | 0.0024 | - | 0.0057 |
| Total effect (Path c: X > Y) | 0.0046 | 0.0028 | -0.0007 | 0.0067 |
| Mediated (a×b/c), % | 27.6 | 15.5 | 100.0 | 15.2 |
